# Missed Opportunities for Nutritional Rehabilitation in Children Admitted to Surgical Wards

**DOI:** 10.1155/2016/3470621

**Published:** 2016-06-27

**Authors:** Pooja Dave, Somashekhar Nimbalkar, Ajay Phatak, Rajendra Desai, Shirish Srivastava

**Affiliations:** ^1^Department of Surgery, Pramukhswami Medical College, Charutar Arogya Mandal, Karamsad, Gujarat 388325, India; ^2^Department of Pediatrics, Pramukhswami Medical College, Charutar Arogya Mandal, Karamsad, Gujarat 388325, India; ^3^Central Research Services, Chautar Arogya Mandal, Karamsad, Gujarat 388325, India

## Abstract

*Background*. Malnutrition in children has serious health and economic consequences. We studied documentation of malnutrition, actual prevalence, and treatment given in children admitted to surgical wards.* Methods*. Retrospective study of 154 patients aged <5 yrs admitted to general surgical, orthopedic, and otorhinolaryngology wards. Records were evaluated for completeness of data, way of documentation, and data quality. Descriptive analysis was done. If malnutrition was not identified and/or proper action was not taken, it was defined as a “missed opportunity.”* Results*. Of 154 records audited, 100 (64.94%) were males, 108 (70.13%) were from general surgery ward, and 78 (50.65%) were residing in suburban area. The mean (SD) age of the study population was 2.32 (1.16) years whereas mean (SD) duration of stay was 5.84 (6.29) days. Weight and height were mentioned in 116 (75.32%) and 8 (5.19%) records, respectively, mostly by nonsurgical personnel. Documentation and treatment of malnutrition were poor. Out of 106 apparently correct weight records, 19 (17.93%) children were severely undernourished and 30 (28.30%) were moderately undernourished whereas 20 (18.87%) children were not undernourished but required nutritional attention.* Conclusion*. There is poor documentation of nutritional indicators of children admitted to surgical wards. From data that was available, it is apparent that malnutrition is at high levels. “Identification” and hence management of malnutrition need more attention.

## 1. Introduction

Approximately 60 million children, under five, accounting for nearly 50% of all India's children are underweight, about 45% children are stunted, and 20% are wasted, suggesting acute malnutrition [1]. Malnutrition among Indian children is twice higher than that in Sub-Saharan Africa and almost five times higher than in China [1]. To improve nutritional status in children under 6 and maternal health, Integrated Child Development Services (ICDS) Scheme was launched in 1975 [2]. Since then, many measures have been taken to combat the condition resulting in a decline in its prevalence. But decline has been slower than what has been achieved in other countries with comparable socioeconomic indicators [3]. Malnutrition contributes significantly to some of the most common causes of mortality in children, in developing countries like India [4].

Malnutrition has been identified in the hospitalized population, up to 25–50% in industrialized as well as developing countries, for more than a decade [5–13]. About 40% of malnutrition was found in Intensive Care Unit (ICU) patients [14] as well as under-five children admitted to pediatric ward [15] in two Indian hospitals. Hospital malnutrition is associated with higher rates of morbidity and mortality [12, 16]. The mortality rate of children with complicated severe acute malnutrition that receive treatment in inpatient setups has remained unacceptably high [17]. Hospital malnutrition is associated with delayed recovery, prolonged hospital stay, and increased healthcare costs and a higher early readmission rate [12, 16–18]. Moreover, deterioration of nutritional status can be precipitated by inadequate detection and intervention during hospitalization [19].

Malnutrition is common among hospitalized children and failure to address it at every contact will place the child at a disadvantage in course of time. It will lead to decreased learning abilities and productivity, increased school dropouts, increased susceptibility to infections, decreased immunity, higher morbidity, and mortality rates in children, which in turn hampers economic strength and human development of the country [1].

There have been few studies which have explored malnutrition in hospitalized pediatric patients in India and none in children admitted to surgical wards. As the nature of training in a surgical department is focused on surgical techniques and postoperative care, while maintaining nutritional status is likely to be taught, we postulated that there would not be any focus on malnutrition presence prior to admission.

Hence we aimed to

1. study the documentation of nutritional parameters and its quality,
2. identify malnutrition prevalence in particular setting and compare with documented malnutrition,
3. demonstrate actual missed opportunities for diagnosis of malnutrition and nutritional rehabilitation by follow-up cases.

## 2. Materials and Methods

An audit of patients' record was performed for patients having following inclusion criteria:

1. Being less than five years of age.
2. Being admitted to the General Surgical/Orthopedic/Otorhinolaryngology Wards of Shree Krishna Hospital, a tertiary care hospital located in rural setup.
3. Being admitted during the period 2009–2013.

In setting of high mortality among children, a large number of deaths occur before 5 years of age [20] and malnutrition contributes to up to 45% of mortality in this age group [21]. Thus we selected children <5 years of age as they make the prime target for malnutrition and nutritional rehabilitation. All the admission records were captured in Microsoft Excel file from this system. Total 980 admissions were identified during the study period. Sample size: assuming 20% compliance with nutritional rehabilitation, we required a sample of size 148 with 90% level of significance and acceptable difference of 5% with finite population correction. The sample size was increased to 185, assuming nonavailability of 25% patient records due to various reasons. A total of 185 admissions were selected randomly from the 980 patients' records using software WINPEPI (http://www.brixtonhealth.com/pepi4windows.html).

This sample size is selected in context to our primary objective to find the missed nutritional rehabilitation opportunities (of documentation, identification, and management if required) and not to calculate the prevalence of malnutrition.

185 records were checked first for written documentation

 (i) of nutritional parameters (including height/length, weight, BMI; any other anthropometrical nutritional indicator, dietary intake, and appetite prior to hospitalization, dietary intake during the hospitalization period, NBM (nil by mouth) duration, and any biochemical profile pertaining to nutrition).

In our tertiary health care center, surgical settings are provided with traditional height and weight measuring devices in both outpatient and inpatient setting. Infantometer and infant weight scale are available upon request.

Meanwhile pediatric outdoor and indoor settings are fully equipped with tools to measure child's length/height, weight, and additional nutritional parameters if required including infantometers, stadiometers, digital weight scales for baby, and traditional height and weight measuring tools. Usually nurses and surgical/pediatric residents measure anthropometry of children.

The 185 records were also checked for

 (ii) assessment done by which hospital department/personnel (which department (anesthesia/pediatrics/surgical) and which staff member (resident/staff nurse) recorded above-mentioned parameters);
 (iii) detailed assessment of nutritional status and diagnosis of malnutrition;
 (iv) the final corrective action carried out for nutritional rehabilitation.

Correct action was defined as (1) dietary counseling to parents, (2) referral to pediatrics department or dietary department and/or referral to PHC (Primary Health Center)/CHC (Community Health Center) during hospitalization or upon discharge, and (3) immediate nutritional management in terms of increased caloric and protein content of hospital diet, vitamin and mineral supplements, and follow-up plan for monitoring nutritional status.

Sociodemographic data (gender, age, and habitat) were audited to find any relationship with malnutrition if any.

Accuracy and reliability of the recorded nutritional data were checked by finding data beyond 4 SD of age limits and finding presence of more than one documented data piece for same parameter, respectively.

Assessment of nutritional status based on mentioned parameters was done, using World Health Organization's growth charts. But as the height/length was not mentioned in most records, weight for age criteria was used to assess the nutritional status. Comparison was made between documented malnutrition diagnosis and the assessed one. If malnutrition was not identified and/or proper action was not taken, it was defined as a “missed opportunity.”

Medical records of patients who had more than one admission were also assessed for their comparative follow-up nutritional status and missed opportunities were actually “demonstrated.”

The study was approved by the institutional ethics committee. Considering the nature of the study, waiver of informed consent was granted.

## 3. Results

Out of 185 admissions included in the study, 154 admission files could be located in the medical records department. Thirty-one files could not be located due to various reasons like medicolegal case, readmission of the patient in the ward, file being used in different study, and so forth. Sociodemographic data of 154 records is shown (Table 1). No significant association was found between malnutrition and sociodemographic profile (gender, habitat, etc.) (Table 2). Of 154 admissions, 18 (11.69%) were infants (<1 year of age). Important data regarding them is mentioned wherever suitable. Out of the 154 admissions audited, weight was mentioned in 116 (75.32%) records and height was mentioned in only 8 (5.19%) records (no length record available for any infant) whereas BMI was not mentioned for even a single record. Further, most of these records were noted in Preanesthesia Checkup or Dietary Assessment Chart filled out by nurses. Only 5 out of 116 weight records were mentioned in surgical case papers. Out of the 8 height records, 4 were mentioned in pediatric case paper and other 4 were mentioned in Preanesthesia Checkup or Dietary Assessment Chart.

Total 10 weight data and 4 height data pieces were found to be spurious (beyond 4 SD limits for the respective age). For example, 55 kg weight and 160 cm height of 3-year-old child were noted suggesting poor and inattentive documentation. Few records had 2 significant different measurements of weight on the same day. Only single record (0.65%) mentioned analysis of weight meaning if underweight for age or not. The similar record also showed analysis of height (0.65%), which was the only record in entire study to show analysis of height. Out of the 106 apparently correct weight records, 19 (17.93%) children were found severely (weight for age <−3*Z*) undernourished and 30 (28.30%) were found moderately (weight for age <−2*Z*) undernourished whereas 20 (18.87%) children were not undernourished but required nutritional attention (<15th centile but >−2*Z*), according to WHO growth chart for weight for age and undernourishment criteria. Out of 14 apparently correct records of 18 infants, 5 infants (27.28%) were moderately undernourished, matching 28.30% moderate malnutrition of main study population. Separate infant severe malnutrition analysis was not possible owing to insufficient or spurious data. “Exclusive breastfeeding” details were not found in any infant's record. Information on appetite was not available in 112 (72.7%), and information regarding dietary intake prior to hospitalization was not available in 113 (73.4%). Nil by mouth (NBM) was mentioned in 108 (70.13%) cases, where NBM means essentially no fluids and food till further order given by surgical personnel before and after surgery. NBM was less than one day in 103 (66.88%), indicating the nutritional deficiency (if any) is chronic and not due to acute illness. Diet advice during hospital stay was not advised in 48 (31.2%) cases. Usually without any diet advice, there is no additional measure taken by hospital pantry. But, when referred to pediatrics or dietitian, they do provide calculation of calories required, necessary diet from hospital (e.g., protein enriched diet, etc.), required vitamins and tonics supplementation, and advice to parents on nutritional rehabilitation. In the remaining 106 (68.8%) cases, these dietary advices were in the form of pre-op/post-op diets by surgeons, regarding their surgeries. These dietary advises were basically instructions of starting/stopping food/oral fluids before and after surgery.

It did not mention the type of diet, nutritive value of diet, and nutritional status of patient. None but only one record (0.65%) had mentioned the diagnosis of low-weight-for-age and referral to dietary department for nutritional rehabilitation.

Out of 154 admissions, 61 patients were identified to have multiple admissions based on unique hospital number. Out of these, 38 patients initial and final admission records were complete for nutritional assessment. Of these 38 patients, 15 (39.47%) lost previous centile weight, 12 (31.58%) gained, and 11 (28.95%) maintained status quo. Further 27 (71.05%) of these 38 patients needed nutritional rehabilitation based on their last admission record.

24, out of 106, admissions (22.64%) were found to be having chronic conditions/conditions affecting nutritional status. The most common diagnoses for the same were, in descending order of their prevalence in study population, esophageal stricture, ureter stricture, diabetes mellitus type 1 (1 patient), cancer (1 patient), gastrectomy (1 patient), unilateral nephrectomy (1 patient), and spina bifida (1 patient). 13 (54%) of these 24 children had malnutrition, which makes contribution of 13 (26.53%) children to total malnourished 49 children. We could not establish any significant correlation between chronic condition and malnutrition status in our study.

## 4. Discussion

The current study showed that most patients were admitted to general surgery ward (70%) which makes it most feasible and applicable site for future intervention, if any. We had 46% of hospitalized children who had malnutrition. This figure is expected as the overall prevalence of malnutrition in the Indian population is high. A similar study carried out in Pune, India, showed 39.8% of children in pediatric ward were malnourished [15]. However most studies of malnutrition conducted in India are done in inpatient settings admitting children, where weight taking and addressing nutritional issues are the norm. We in the current study show that only five out of 154 case papers mentioning weight were made by surgical personnel. Missing, inconsistent, and spurious documentation is the key finding of this study that suggests inattentiveness and lack of awareness towards the importance of nutrition in health of children as well as burden of malnutrition. Though we do realize the poor feasibility of measuring weight by surgeons themselves, lack of assessment of nutritional status in all cases shows that malnutrition is not on the radar of the admitting surgeons. This has serious implications on management as wound healing and recovery from surgery are delayed in patients with poor nutritional status. Malnutrition has been seen to be associated with higher mortality, higher morbidity, and increased length of stay, increased costs, and higher need for readmission [12, 16]. Prospective studies in specialized settings which have evaluated nutritional status for its role in recovery have seen malnutrition in 53–59% of admitted patients. However, in these specialized settings it is likely that the underlying disease contributed further to the undernutrition and hence a large proportion of patients had malnutrition [22, 23]. A systematic review on malnutrition in adult admitted patients in the UK and European countries demonstrated that more than half of patients at risk for malnutrition were missed and hence did not receive care relevant to nutrition [24]. Unfortunately not many pediatric studies are available in this subject especially highlighting nutritional rehabilitation, nutritional status assessment for children in surgical wards before and after surgery, and the nutritional implications for children who have surgery particularly if it involves the mouth and/or GIT.

These missed opportunities ought to be addressed by nutritional screening on admission by ancillary staff. There are various tools for screening in children admitted to hospital such as the Pediatric Yorkhill Malnutrition Score (PYMS), Screening Tool for the Assessment of Malnutrition in Pediatrics (STAMP), and Pediatric Subjective Global Nutritional Assessment (SGNA) [25]. However these have been developed in resource replete countries and there is a need to validate their accuracy in setting with high prevalence of malnutrition before they can be recommended for routine use. The Malnutrition Assessment tool should be administered in each ward during admission as well as discharge. This will monitor preadmission nutritional status, change in it during hospitalization, end nutritional status, and follow-up improvement/deterioration if any. It can be simplified by checklists and algorithms to follow standardization in diagnosis of malnutrition as well as nutritional rehabilitation management. Pediatric patient presenting with malnutrition can be referred to pediatrician and/or nutritionists for further evaluation and elaborative management. These models need to be implemented with continued monitoring to assess their effectiveness. As the study data shows 39.5% of patients who had a second admission declined to a lower weight centile while 28.9% maintained the same centile. The weight loss on subsequent admissions indicates actual missed opportunities of diagnosis of malnutrition as well as possible nutritional rehabilitation. Failure of nourishment in between successive hospital admissions may suggest failure of government schemes for malnutrition too. This is likely due to the fact that the factors contributing to malnutrition were persistent. Efforts to identify and rectify nutritional status need to be carried out but may not be useful as reasons for malnutrition are beyond the realm of medical care.

Further studies can be done on application and efficacy of nutritional screening tools, early recognition of hospital malnutrition, rate of references to pediatricians after tool application, and treatment of the same. While resolving malnutrition in a developing country may be a moving goalpost, using checklists to screen for malnutrition and rehabilitative management need to be explored to improve the health of the admitted children.

The current study shows the lack of attention towards nutritional status of pediatric patients admitted in surgical wards, which are responsible for “missed opportunities of diagnosis of malnutrition and nutritional rehabilitation.” More attentive approach of nonpediatric specialties towards pediatric patients would help in reducing burden of hospitalized malnutrition as well as general malnutrition. Screening tools and checklists may be explored to simplify the diagnosis and management process and should be a part of routine hospitalization process to demonstrate its widespread effects. The core message here nonetheless would be “treat the patient and not just the disease.” Holistic approach should be the guiding principle of medical practice.

## Figures and Tables

**Table 1:** Characteristics of the study population.

Particulars	Frequency (%)
Gender	
Male	100 (64.94)
Female	54 (35.06)

Habitat	
Urban	36 (23.38)
Rural	40 (25.97)
Suburban	78 (50.65)

Ward	
General surgery	108 (70.13)
Orthopedics	32 (20.78)
Otorhinolaryngology (ENT)	14 (9.09)

Age	
Mean (SD) (range)	2.32 (1.16) (29 days to 4 years)

Duration of stay	
Mean (SD) (range)	5.84 (6.29) (1 to 37 days)

**Table 2:** Malnutrition and associated sociodemographic profile.

	Moderate malnutrition	Severe malnutrition	Total malnutrition
Gender			
Male	22%	14.8%	36.8%
Female	14%	18.5%	32.5%

Habitat			
Urban	13.9%	16.7%	30.6%
Suburban	22.5%	12.5%	35%
Rural	20.5%	16.7%	37.2%

Ward			
Surgery	16.7%	17.6%	34.3%
Orthopedics	34.4%	6.3%	40.7%
ENT	7.1%	21.4%	28.5%
